# Commentary: Trem2 Deletion Reduces Late-Stage Amyloid Plaque Accumulation, Elevates the Aβ42:Aβ40 Ratio, and Exacerbates Axonal Dystrophy and Dendritic Spine Loss in the PS2APP Alzheimer's Mouse Model

**DOI:** 10.3389/fnagi.2020.00219

**Published:** 2020-08-18

**Authors:** Sachchida Nand Rai, Vivek K. Chaturvedi, Brijesh Kumar Singh, Mohan P. Singh

**Affiliations:** ^1^Centre of Biotechnology, University of Allahabad, Prayagraj, India; ^2^Department of Pathology and Cell Biology, Columbia University Medical Centre, Columbia University, New York, NY, United States

**Keywords:** Alzheimer's disease, knockout, mouse model, TREM2 (triggering receptor expressed on myeloid cells), amyloid, tau, transgenic mice, axonal dystrophy

Accumulating pieces of evidence have shown that microglia have both protective as well as detrimental impacts depending upon the stage of the disease (Hammond et al., 2019). A number of mutations have been reported in microglial genes that are linked with increased AD risk. The triggering receptor expressed on myeloid cells 2 (Trem2) is one of the most critical AD risk genes found in microglia. In animal models and human studies as well, several experiments have been performed which showed that Trem2 and its variants effectively modulate the function of microglia. Trem2 effectively alters the microglial functions such as activation, proliferation, phagocytosis, inflammation, survival, and metabolism.

## Introduction

In this article, the authors explored the role of Trem2 in PS2APPAD mouse model. Authors compared PS2APP transgenic mice vs. Trem2 knockout PS2APP mice (PS2APP;Trem2ko) at different points of age. Authors suggested that the toxic activity of Aβ species in neurons is reduced because of its compaction by Trem2 into dense plaques. Thus, this Trem2-dependent compaction of Aβ into dense plaques shows neuroprotective activity. In Trem2-deficient mouse, the effect of plaque load varies across the models, age, and the region of the brain being analyzed (Jay et al., 2017; Parhizkar et al., 2019). On other hand, in Trem2-deficient Alzheimer disease (AD) mice, status of neurofibrillary tangles remains uncertain; it may either increase or decrease (Jay et al., 2015; Wang et al., 2016). Similarly, human tau protein studies also show variable responses on deletion on Trem2. Unlike mentioned above, Trem2 deletion is protective in thePS19 model of AD and reduces synaptic loss specifically in hippocampus and entorhinal cortex and also rescues the brain atrophy (Yoshiyama et al., 2007; Leyns et al., 2017).

## Discussion

In this article, to prevent the possibility of biasness, authors have explored the impact of Trem2 deletion on microglial activation, plaque accumulation, and neuronal pathology in the PS2APP model at various ages and in both the sexes (Meilandt et al., 2020). With increasing age, female mice accumulate more amyloid plaques and also showed prominent gliosis as compared to that in male PS2APP mice. On the contrary, the accumulation of plaque was significantly reduced at older ages in both female and male PS2APP;Trem2ko as compared to PS2APP mice. Total amyloid plaques are generally reduced in older ages. Besides this, at older ages, neuronal dystrophy and histopathology were well-observed in PS2APP;Trem2ko mice, which showed an enhanced ratio of Aβ42:Aβ40. Furthermore, in cerebrospinal fluid (CSF) of PS2APP;Trem2ko mice at approximately 12 months of age, the ApoE-laden microglia, enhanced levels of soluble fibrillar oligomeric Aβ, and increased neurofilament light chain (NFL) were found. The level of proinflammatory factors related to Wnt-related signaling was also reduced as shown by transcriptome analysis of PS2APP;Trem2ko mice. Transcriptome analysis showed that disease/damage-associated microglial (DAM) activation found in the AD model is Trem2-dependent (Keren-Shaul et al., 2017; Krasemann et al., 2017). Still, there is controversy regarding the protective or detrimental role of DAM in different neurodegenerative diseases (Keren-Shaul et al., 2017; Krasemann et al., 2017).

The level of plaques is slightly more in PS2APP;Trem2ko females at 6 to 7 months of age but reduced in both sexes of mice at later ages. At the early stage of pathology, the formation and seeding of plaques were reduced in PS2APP;Trem2ko mice, which are dependent on Trem2. These mice showed such activity by uptake and degradation of soluble Aβ species at an early stage while promoting the sequestration of Aβ into the existing formed plaques at later stages. Microglial clustering around Aβ plaques were also reduced in both sexes of all ages in PS2APP;Trem2ko mice. This study showed that plaque-proximal dendritic spine loss was more aggravated in PS2APP;Trem2ko mice. Thus, for neurons, Trem2-dependent microglial activation around plaque is protective, which is vastly different than other complement-mediated pathways by microglia (Shi et al., 2017; Wu et al., 2019). This study also showed that with age the ratio of Aβ42:Aβ40 was decreased mostly in the insoluble fraction, which is responsible for the more compact plaques in thePS2APP mice brain. In the soluble fraction, enhanced levels of fibrillar oligomeric Aβ have been found, which is ultimately responsible for the evident neuronal injury in these AD models.

The beneficial impact of preventing AD progression and cognitive decline by inhibiting the formation and accumulation of Aβ is quite questionable, as several studies along with some clinical trials have failed to demonstrate it (Morris et al., 2014; Itzhaki et al., 2016). Some researchers also have suggested that chronic microglial activation causes significant neurotoxicity in AD (Heneka et al., 2015; Park et al., 2018).

This article shows contrasting findings; the pathologies related to axonal dystrophy and dendritic spine loss become more detrimental by preventing the microglial response to Aβ pathology via Trem2 deletion. Also, in CSF, the level of NFL was enhanced, which is a potent biomarker in AD along with other neurodegenerative diseases. Microglial clustering around plaques was significantly impaired, and plaques were more diffuse in PS2APP;Trem2ko as compared to its wild-type counterpart. In Trem2-deficient brains, the ratio of Aβ42:Aβ40 and the amount of soluble and fibrillar Aβ oligomers were considerably elevated. Therefore, the compaction of Aβ into dense plaques is a protective microglial activity, which is Trem2-dependent, which reduces the exposure of neurons toward toxic Aβ species (Meilandt et al., 2020).

## Conclusion

The authors concluded that the form of β-amyloid is far more dangerous as compared to the amount in the brain. In this finding, microgliosis, which was Trem2-dependent, caused the compaction of β-amyloid into less damaging form. Hence, to prevent the progression of AD, therapeutics that enhance microgliosis along with amyloid content show convincing findings.

Authors should clearly study the complete correlation between the two forms of microglia. Besides, the role of Trem2 should also be studied in a chemical-induced AD model to correlate these findings with PS2APP;Trem2ko mice. Thus, further study will be needed to evaluate the mechanism of action behind Trem2 in AD.

## Author Contributions

SR, BS, and VC co-wrote the manuscript. MS edit the whole manuscript. All authors contributed to the article and approved the submitted version.

## Conflict of Interest

The authors declare that the research was conducted in the absence of any commercial or financial relationships that could be construed as a potential conflict of interest.
